# Supplementary material for: Targeting of the NOX1/ADAM17 Enzymatic Complex Regulates Soluble MCAM-Dependent Pro-Tumorigenic Activity in Colorectal Cancer
Source: Biomedicines. 2023 Nov 30;11(12):3185. doi: 10.3390/biomedicines11123185 (PMC10740863; doi:10.3390/biomedicines11123185)
Supplement: Supplementary file 1 [file biomedicines-11-03185-s001.zip › Supplementary Figure legends.pdf]

Supplementary Figures legend

Supplementary Figure S1: ADAM17 pharmacological inhibition does not change lymphocyte content in MC38 tumors

(A-D) $CD4^+$ (A), $CD8^+$ (B) T lymphocytes, B lymphocytes ($CD3^-$/$B220^+$) (C), and Natural killer T lymphocytes ($CD3^+$/$NK1.1^+$) (D) of MC38 tumors were analyzed by flow cytometry for the corresponding treated groups. (E) Tumor pan macrophages ($CD45^+$/$CD11b^+$/$F4/80^+$) (left) and tumor-associated macrophages (TAM) ($CD45^+$/ $CD11b^+$/$CD68^+$/$F4/80^+$) (right) in MC38 tumors were analyzed by flow cytometry for the corresponding treated groups.

Supplementary Figure S2: Expression of *NOX1*, *ADAM17*, and *MCAM* in CRC correlates with shorter relapse-free survival, advanced tumor stage, and *KRAS* mutations

(A-B) Kaplan–Meier survival analysis of the index of *NOX1 (A),* and *ADAM17 (B)* mRNA expression (high [red] *vs.* low [blue]) relative to the median value in CRC patient tumor samples. (C) Expression of *NOX1* (left), *ADAM17* (middle), and *MCAM* (right) mRNA in CRC patient tumor samples according to tumor stages (1 to 4). (D) Expression of *NOX1* (left), *ADAM17* (middle), and *MCAM* (right) in *KRAS* mutated and wild-type patients from CRC patients.

Supplementary Figure S3: Characterization of MCAM expression on Human Dermal Lymphatic Endothelial Cells (HDLEC)

(A) Specific mouse and human mRNA expression strategies in tumors were explored by Nanostring arrays using specifics mouse and human probes. (B-D) MCAM membrane expression in HDLEC cells was analyzed by flow cytometry (B) and fluorescent microscopy (C) using a specific anti-MCAM antibody (Sendo-1) or corresponding control antibody (IgG). Fluorescence intensity of membrane MCAM expression by flow cytometry (B) and fluorescent microscopy pictures were shown (C).
