# Supplementary figures and images for: Targeting of the NOX1/ADAM17 Enzymatic Complex Regulates Soluble MCAM-Dependent Pro-Tumorigenic Activity in Colorectal Cancer
Source: Biomedicines. 2023 Nov 30;11(12):3185. doi: 10.3390/biomedicines11123185 (PMC10740863; doi:10.3390/biomedicines11123185)

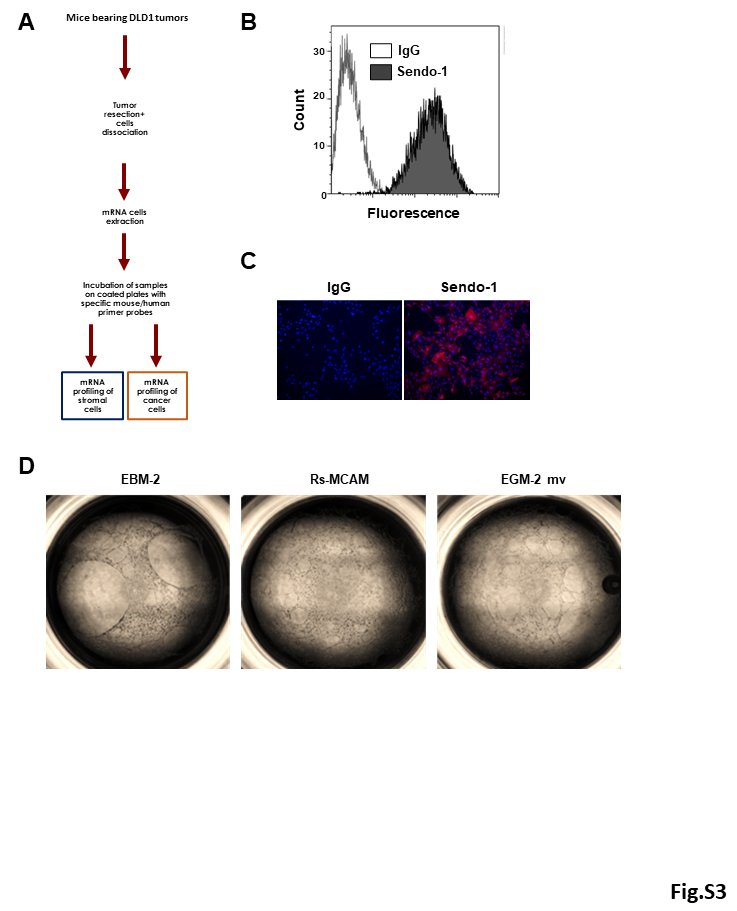

Supplement: Supplementary file 1 [file biomedicines-11-03185-s001.zip › Figure S3.tif]

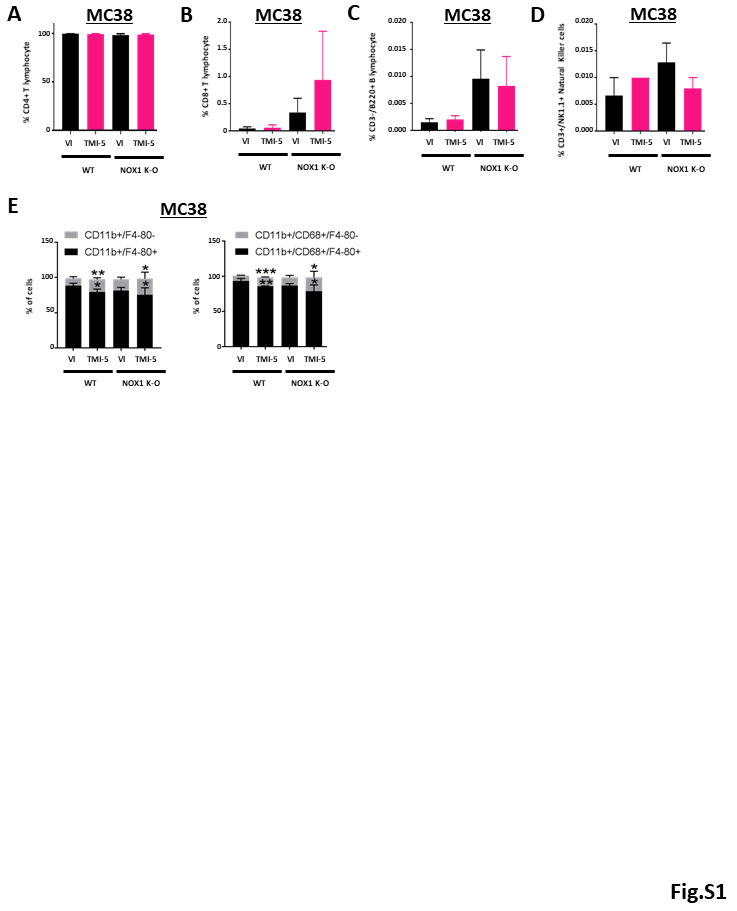

Supplement: Supplementary file 1 [file biomedicines-11-03185-s001.zip › Supplementary Figure S1.tif]

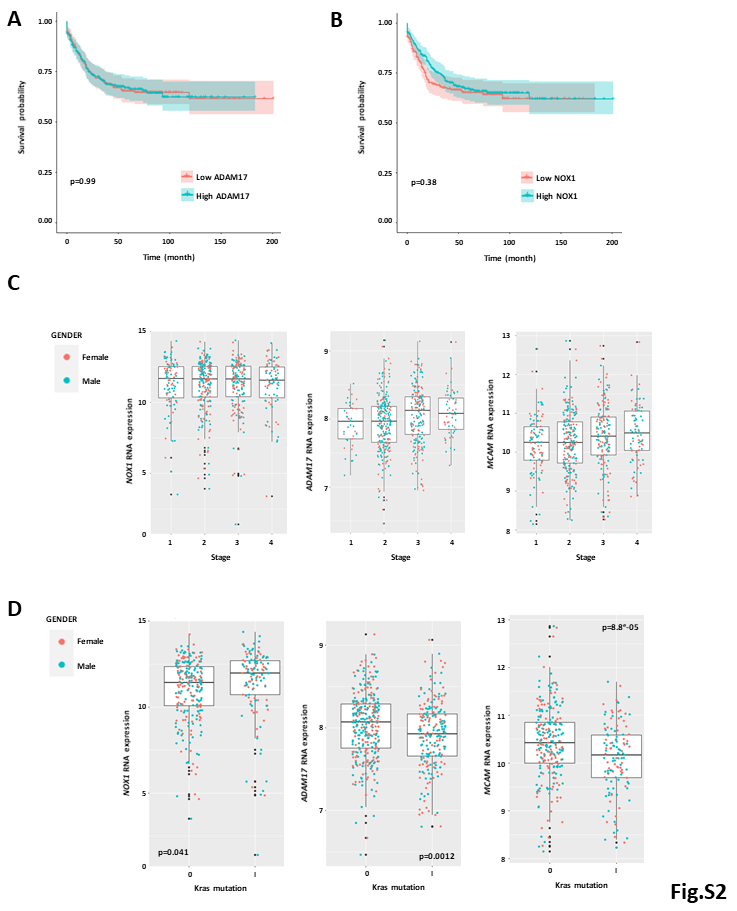

Supplement: Supplementary file 1 [file biomedicines-11-03185-s001.zip › Supplementary Figure S2.tif]
